# Neurofilament Biomarkers in Neurology: From Neuroinflammation to Neurodegeneration, Bridging Established and Novel Analytical Advances with Clinical Practice

**DOI:** 10.3390/ijms26199739

**Published:** 2025-10-07

**Authors:** Ariadne Daponte, Christos Koros, Charalampos Skarlis, Daphne Siozios, Michail Rentzos, Sokratis G. Papageorgiou, Maria Anagnostouli

**Affiliations:** 1 Neuromuscular Diseases Unit, First Department of Neurology, School of Medicine, National and Kapodistrian University of Athens (NKUA), Aeginition University Hospital, Vas. Sofias 72-74, 11528 Athens, Greece; ariadnidaponte@gmail.com (A.D.); mrentzos@med.uoa.gr (M.R.); 2 Neurodegenerative Diseases and Rare Dementias Unit, First Department of Neurology, School of Medicine, National and Kapodistrian University of Athens (NKUA), Aeginition University Hospital, Vas. Sofias 72-74, 11528 Athens, Greece; chkoros@gmail.com (C.K.); sokpapa@med.uoa.gr (S.G.P.); 3 Multiple Sclerosis and Demyelinating Diseases Unit, Center of Expertise for Rare Demyelinating and Autoinflammatory Diseases of CNS, First Department of Neurology, School of Medicine, National and Kapodistrian University of Athens (NKUA), Aeginition University Hospital, Vas. Sofias 72-74, 11528 Athens, Greece; charskarlis@med.uoa.gr; 4 Medical Degree English Program, Athens School of Medicine, National and Kapodistrian University of Athens (NKUA), 10679 Athens, Greece; dsiozios@gmail.com

**Keywords:** neurofilaments, biomarkers, multiple sclerosis, amyotrophic lateral sclerosis, Alzheimer’s disease, Parkinson’s disease, neuroinflammation, neurodegeneration, immunotherapies

## Abstract

Neuroaxonal damage underlies permanent disability in various neurological conditions, both neuroautoimmune and neurodegenerative. It is crucial to accurately quantify and monitor axonal injury using biomarkers to evaluate disease progression and treatment effectiveness and offer prognostic insights. Neurofilaments (NFs), and especially neurofilament light chain (NfL), show promise for this purpose, as their levels increase with neuroaxonal damage in both cerebrospinal fluid and blood, independent of specific causal pathways. Recent advances in ultrasensitive immunoassays enable the reliable detection of NFs in blood, transforming them from research tools into clinically applicable measures. In multiple sclerosis (MS), serum NfL correlates with disease activity, treatment response, and long-term disability, and may complement MRI in monitoring subclinical progression. In MS, NfL is primarily emerging as a marker of disease activity and treatment response; in amyotrophic lateral sclerosis (ALS), it has progressed further, being integrated into clinical trials as a pharmacodynamic endpoint and considered by regulatory agencies as a drug development tool. Additionally, NFs are increasingly being investigated in Alzheimer’s disease, frontotemporal dementia, and other neurodegenerative disorders, though their disease specificity is limited. Ongoing challenges include older and novel assay harmonization, normative range interpretation, biological and analytical variability, and integration with other molecular and imaging biomarkers. This critical narrative review synthesizes the existing literature on NFs as diagnostic, prognostic, predictive, and pharmacodynamic biomarkers and discusses their role in therapeutic development and precision medicine in neuroautoimmune and neurodegenerative diseases.

## 1. Introduction

Neuroaxonal damage is one of the central pathological processes in numerous neurological disorders, and it usually underlies permanent disability. Therefore, an accurate quantification and a longitudinal follow-up of neuroaxonal damage are crucial to the assessment of progression and response to treatment, in addition to providing prognostic insights.

Neurofilaments (NFs) have been thoroughly studied and are recently promising biomarkers, particularly as they increase in cerebrospinal fluid (CSF) and blood, reflecting neuroaxonal injury. With technological advancement, new assays with high sensitivity have been developed, and they allow reliable measurement of NFs. These assays have also opened new opportunities to study neurofilament biology and its translational application in neurological disorders.

### 1.1. Structure of Neurofilaments and Their Role in Healthy Neurons

NFs are structural proteins located in the cytoplasm of neurons. They are composed of 10 nm large filaments (10 nm), with a diameter intermediate between actin (6.5 nm) and microtubules (25nm), belonging to the class of intermediate filaments (IF) [1]. In the central nervous system (CNS), they assemble with α-internexin, whereas in the peripheral nervous system (PNS), they associate with peripherin [2,3]. NFs are heteropolymers and they are composed of different isoforms named according to their molecular weight: neurofilament light (NfL), medium (NfM), and heavy chains (NfH). Each subunit consists of a N-terminal head domain, a highly conserved α-helical rod domain, and a C-terminal tail domain of variable length [1].

NFs also undergo extensive posttranslational modifications, including phosphorylation, O-linked glycosylation, nitration, and ubiquitination. These modifications modulate their assembly and spacing as well as their interactions with other cytoskeletal elements [3].

**Figure 1:**
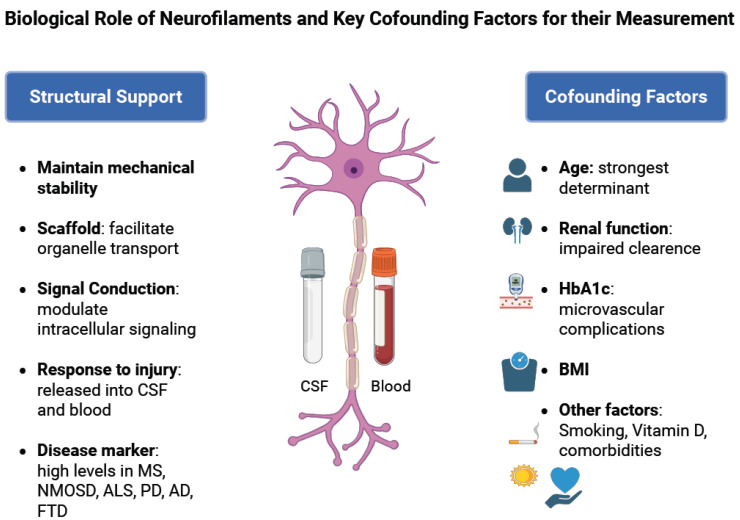
Biological role of neurofilaments and key confounding factors for their measurement.

NFs are most abundant in large myelinated axons, but they can also be found in smaller amounts in cell bodies, dendrites, and synapses [3,4]. They have several biological functions within the nervous system (Figure 1). Their best-known role is mechano-resistance. Their structural meshwork offers mechanical robustness to long axons, allowing them to withstand mechanical insults and to maintain adaptability to environmental stress [5]. NFs serve as a scaffold for the spatial organization and transport of organelles, including mitochondria and endoplasmic reticulum, and they help stabilize microtubule networks. Additionally, they play a significant role in the regulation of the radial growth of large fast-conducting neurons [2,6]. Overall, NFs are key players in neurotransmission and signaling, in regulating nerve conduction, and in contributing to cytoskeleton dynamics [1,5].

Neurofilaments (NFs) provide structural support and mechanical stability to axons, serve as scaffolds for organelle transport, and modulate intracellular signaling and conduction. Following axonal injury, NFs are released into the cerebrospinal fluid (CSF) and subsequently into the blood, where elevated levels act as a marker of neuroaxonal injury in diseases such as multiple sclerosis (MS), neuromyelitis optica spectrum disorder (NMOSD), amyotrophic lateral sclerosis (ALS), Parkinson’s disease (PD), Alzheimer’s disease (AD), and frontotemporal dementia (FTD). Several physiological and metabolic factors may confound NfL measurement, including age (the strongest determinant), renal function (impaired clearance), HbA1c (microvascular complications), body mass index (BMI), and other variables such as smoking, vitamin D status, and comorbidities.

### 1.2. Role of Neurofilaments in Diseases with Axonal Injury

**Figure 2:**
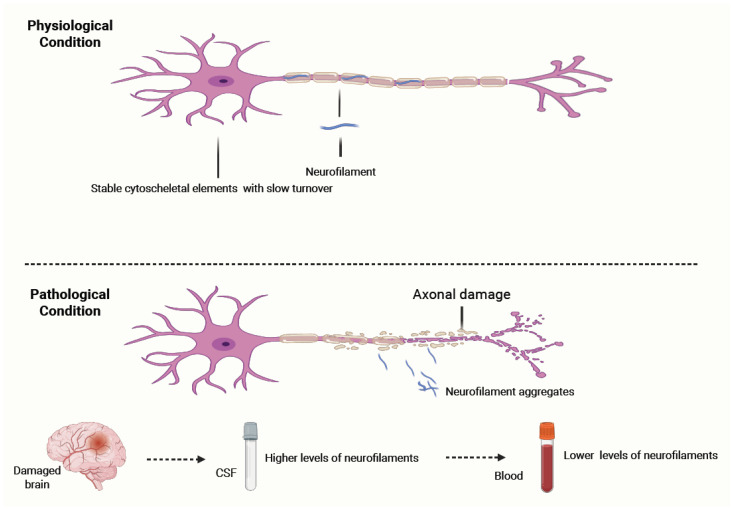
The role of neurofilaments under physiological and pathological conditions.

Under normal conditions, NFs are stable cytoskeletal elements with a slow turnover. The importance of NFs becomes most evident under pathological conditions. Their aggregation is a marker of neuroaxonal injury, and they are significantly higher in the CSF and blood of patients with various neurological diseases characterized by axonal injury of myelinated neurons [7,8,9,10,11]. NFs are passively released into CSF in response to neuroaxonal pathology, regardless of whether it is due to inflammatory, traumatic, ischemic, or degenerative mechanisms [11,12,13]. This makes NFs essentially an unspecific marker of axonal damage rather than a pathology-specific marker, such as, for example, phosphorylated Tau in Alzheimer’s Disease [14]. In clinical practice, though, NFs can be quite useful in differentiating different conditions with a higher versus a lower rate of neuron degeneration [3] (Figure 2).

In this review, the term neurofilaments (NFs) is used when studies have measured more than one isoform (e.g., NfL and pNfH) or have reported results without specifying a subunit. Neurofilament light chain (NfL) is referred to explicitly when studies focus on this isoform, which is by far the most extensively studied and validated in clinical practice. This distinction is important, as NfL has emerged as the leading candidate for routine biomarker implementation, while other subunits, such as pNfH, may provide complementary but less consistently replicated information.

- Under physiological conditions, neurofilaments (NFs) are stable cytoskeletal components with slow turnover, maintaining axonal structure and integrity.
- In response to axonal injury caused by inflammatory, traumatic, ischemic, or degenerative mechanisms, NFs aggregate and are released into the cerebrospinal fluid (CSF) and subsequently into the blood. Their levels correlate with the extent of axonal damage and can remain elevated for weeks to months before clearance. NF concentrations are significantly higher in CSF than in blood, with a typical gradient of ~1:40. Clinically, NF measurement serves as a sensitive, though non-specific, biomarker of neuroaxonal injury and can help differentiate conditions with higher versus lower rates of neuronal degeneration.

NF levels rise in direct proportion to the extent of axonal injury and remain elevated for several weeks to months before clearance. NF levels in the CSF are higher and more reflective of CNS injury. From the CSF, NFs can cross into the bloodstream, where they are detectable at much lower concentrations, usually with a concentration gradient of about 1:40 relative to the CSF [3,14]. NF degradation and clearance are regulated by various molecular mechanisms, including proteolysis, autophagy, renal excretion, and possibly uptake by glial or immune cells; however, these mechanisms remain incompletely understood [4,5,15].

The intermediate filament (IF) family comprises 70 tissue-specific genes that provide a cytoplasmic network in various tissues [16]. Genes controlling NF structure and regulatory pathways have been identified as the genetic cause of more than 80 human diseases, reflecting the biological importance of IF [17]. Types of Charcot–Marie–Tooth (CMT) disease, spastic ataxia, and hereditary spastic paraplegia (HSP) are some of the neurological diseases caused by genetic mutations on NFs [5]. A combination of fundamental and disease-oriented research on NFs is needed in order to comprehend their diverse composition and heterogeneity, as well as how they regulate signaling and neuronal physiology, and how they are implicated in disease pathophysiology [5].

### 1.3. Confounding Factors Influencing Nf Levels

Interpretation of NF concentrations, particularly serum NfL, requires careful consideration, as there are several confounders that can influence them (Figure 1). Age is the most consistent and potent determinant. NF levels increase by approximately 2–3% per year, but that rate accelerates even more beyond the age of 60, potentially reflecting subclinical comorbid pathology [18,19,20]. A recent population-based study of 1706 participants from the US National Health and Nutrition Examination Survey (NHANES) provides a comprehensive quantification of a variety of confounders in neurologically healthy individuals. Using multivariable models, they evaluated the predictive capacity of 52 demographic, lifestyle, comorbidity, anthropometric, or laboratory characteristics that could explain the variability of sNfL levels [19]. The most prominent contributors that were positively predictive of elevated sNfL levels were age, creatinine, and HbA1c. Associations with renal function markers reinforce previous findings that impaired clearance may elevate NfL levels, while the link with HbA1c is likely associated with microvascular complications such as diabetic neuropathy or cerebrovascular disease [19]. BMI showed a weak inverse association, consistent with European cohort data, but was not a primary driver of variability after accounting for metabolic and renal indices [19].

Other studies have found that BMI, as well as other additional factors, may influence NfL levels, such as smoking, Vitamin D levels, and non-neurological diseases (e.g., atrial fibrillation, heart failure, and hypertension) [18]. To date, the literature has been fully consistent in that sNfL is not sex-dependent [18,21]. Overall, these results highlight that sNfL must be interpreted against appropriately adjusted reference ranges based on demographics and comorbidities, especially age, renal function, and metabolic health.

## 2. Methods of NfL Measurement

In clinical research, the most extensively studied isoform is neurofilament light (NfL). NfL levels are found consistently increased in CSF (cNfL) and blood (serum or plasma, sNfL) in a wide spectrum of diseases, including multiple sclerosis (MS), traumatic brain injury, amyotrophic lateral sclerosis (ALS), and other neurodegenerative disorders, compared with age-matched controls [22,23]. The methods for NfL quantification in biological fluids have advanced significantly over the past decade, supporting its transition from a research tool to a viable clinical biomarker as seen through multiple applications.

### 2.1. Biological Sources of NfL

Following axonal injury, NfL is released into the CSF and subsequently enters the bloodstream, typically in much lower amounts (≈2% of CSF levels) [14,18]. Historically, CSF has been the primary fluid used for NfL measurement due to its direct contact with the CNS, providing high sensitivity for early detection. However, the invasive nature of lumbar puncture, along with the need for routine and longitudinal monitoring in clinical practice, has driven the shift toward blood-based quantification of NfL (sNfL) as a more ideal solution [24,25]. On the other hand, measurement of NfL levels in blood offers a more practical, minimally invasive alternative method with high correlation to CSF levels, which is suitable for repeated testing and highly relevant in clinical settings. Paired studies indicate that after acute brain injury, NfL levels in CSF and blood follow similar dynamics. NfL levels peak at 40–70 days and return toward baseline within approximately six months [26]. Overall, sNfL has been established as the main method for NfL measurement, although some uncertainty remains about whether this equivalence holds across all concentrations and disease subgroups. For example, in conditions with both CNS and PNS neuronal injury, such as ALS, interpreting blood measures in terms of these distinct pathologies may be ambiguous [18].

### 2.2. Analytical Platforms for NfL Quantification

**Figure 3:**
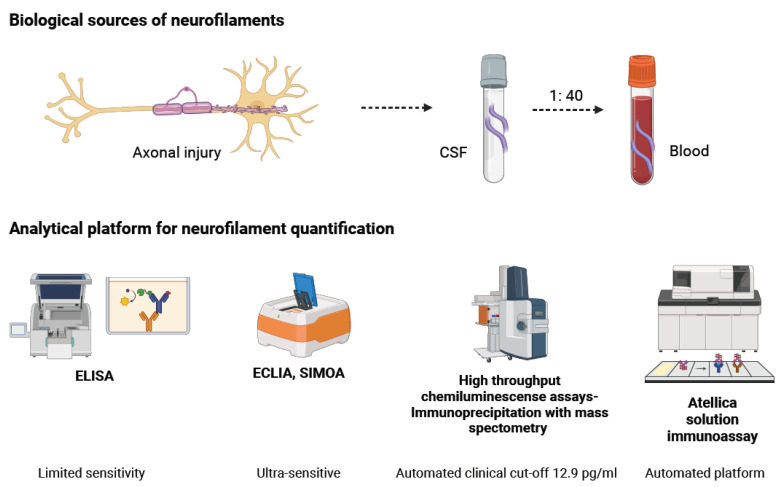
Biological sources and analytical platforms for NfL quantification. Following axonal injury, neurofilaments (NfL) are released into the cerebrospinal fluid (CSF) and subsequently into the blood, where they can be measured at lower concentrations. Several immunoassay platforms are available for NfL quantification: (i) enzyme-linked immunosorbent assay (ELISA), the earliest method with limited sensitivity mainly applied to CSF; (ii) electrochemiluminescence (ECLIA) and single molecule array (SIMOA), which provide ultrasensitive detection suitable for both CSF and blood; (iii) a novel Atellica solution immunoassay, a high-throughput chemiluminescent immunoassay enabling automated large-scale clinical testing; and (iv) immunoprecipitation combined with mass spectrometry, an emerging technology allowing detection of NfL proteoforms and distinction of CNS versus PNS pathology. CNS: central nervous system; PNS: peripheral nervous system.

A variety of immunoassay platforms have been developed, each with distinct advantages and limitations (Figure 3 and Table 1).

Enzyme-linked immunosorbent assay (ELISA): ELISA is the earliest method developed for NfL detection and uses antibodies targeting the mid-domain rod region of the protein [27]. This method is mainly applied to CSF due to its limited sensitivity in peripheral blood. While effective for research purposes, traditional ELISAs are labor-intensive and unsuitable for clinical scalability [28].

Electrochemiluminescence (ECLIA): ECLIA has been introduced as a semi-sensitive method that can detect NfL in both CSF and serum. It offers partial automation and is more feasible for medium-throughput settings but remains less widely used than newer platforms [19].

Single molecule array (SIMOA): SIMOA is an ultra-sensitive immunoassay technology capable of detecting NfL at sub-picogram concentrations with a strong correlation between CSF and serum NfL levels. It offers up to 126-fold and 25-fold higher sensitivity over ELISA and ECLIA assays, respectively [29]. In 2022, the FDA granted breakthrough device designation to the Quanterix Simoa^®^ NfL plasma test as a prognostic tool for relapsing-remitting multiple sclerosis [18].

High-throughput chemiluminescent immunoassays: Platforms such as the Siemens Atellica^®^ integrate NfL testing into routine clinical chemistry analyzers, enabling full automation with robust reproducibility. For instance, a serum NfL cutoff of 12.9 pg/mL has been suggested as a clinically meaningful threshold in MS [25]. This NfL essay is an approved prognostic risk assessment test setting a new standard, with a clear decision threshold, and it delivers innovative, reproducible results for improving MS management. Additionally, it easily consolidates into the clinical laboratory workflow, as it is available for both serum and plasma [23,30].

Emerging technologies: Novel methods such as immunoprecipitation using mass spectrometry are beginning to characterize NfL proteoforms in biological fluids. These studies suggest that CSF contains truncated NfL fragments (both N- and C-terminal) rather than full-length protein. Brain tissue, on the other hand, predominantly contains intact NfL [31]. A better understanding of these species may open avenues for assays that can distinguish CNS-specific versus PNS-specific pathology by targeting proteins such as α-internexin (CNS) or peripherin (PNS) [18].

### 2.3. Pre-Analytical Considerations

Despite improvements in assay technology, pre-analytical and physiological factors continue to play a role in NfL measurement and interpretation.

- Sample handling: Studies have shown that appropriate storage, typically at −80 °C, is essential to maintain protein stability, particularly by avoiding repeated freeze–thaw cycles. The type of collection tube does not significantly influence NfL levels [32].
- Physiological confounders: NfL levels increase with age and are influenced by renal function, metabolic status (e.g., HbA1c), and other comorbidities. Age-adjusted reference values and awareness of comorbidities are essential when interpreting NfL results in both healthy individuals and patients with neurological disease [19]. Even though most laboratories only use age-adjusted reference values, prediction models have been developed and published so that they can be used for patients with such confounders [19,33].
- Comparison of different assays: Different assays, like SIMOA and chemiluminescent immunoassays, correlate well with each other. However, it should be taken into account that the absolute NfL levels are not identical, and are thus not comparable [34]. Specialized conversion models have been developed and can be utilized for comparing NfL levels by different assays, for example, in patients with longitudinal NfL measurements [19,34].

## 3. Neurofilaments in Multiple Sclerosis

### 3.1. Introduction to Multiple Sclerosis

Multiple sclerosis (MS) is a chronic immune-mediated disorder of the central nervous system (CNS), characterized by inflammation, demyelination, and axonal degeneration of various degrees. It usually manifests between the ages of 20 and 40 years, and it stands as the leading cause of disability in young adults. MS is a clinically heterogeneous disease with four distinct subtypes: relapsing-remitting (RRMS), secondary progressive (SPMS), primary progressive (PPMS), and relapsing-progressive (RPMS). In the last two decades, two other subtypes, at the ends of the age spectrum, have been well recognized: pediatric-onset (POMS) and late-onset (LOMS) forms. Even though the exact underlying cause of MS remains elusive, current evidence suggests that MS arises from a complex interaction of genetic predisposition, environmental influences, and hormonal and epigenetic factors [35,36]. This interaction leads to both focal inflammatory demyelination and diffuse neurodegenerative processes.

The management of MS necessitates continuous assessment of disease activity and optimizing treatments. Over the past two decades, substantial advances have been achieved in the management of MS. Numerous highly efficacious immunomodulatory treatments are now available, and some can alter the disease course and slow disability accumulation [37,38]. Nonetheless, there is an unmet need for reliable biomarkers in order to provide insight into disease activity, therapeutic efficacy, and prognosis across the entire MS spectrum.

Due to the pathophysiological complexity and variable clinical nature of this disease, establishing reliable biomarkers has proven to be quite the challenge. While neuroimaging and immunogenetics have significantly advanced, they often fail to capture subtle, diffuse neuroaxonal injury or ongoing disease activity in the absence of new MRI lesions. This is especially relevant given that progression independent of relapse activity (PIRA), which refers to disability accrual in the absence of relapses and inflammatory activity, may occur even in patients without clinical relapses [39]. Accordingly, there is a growing interest in dynamic and minimally invasive fluid biomarkers capable of capturing real-time CNS damage [24,28]. Among these, neurofilament light chain (NfL) has emerged as the most promising candidate. With the emergence of ultrasensitive assays, serum NfL (sNfL) has become a reliable and accessible biomarker, enabling more personalized MS care [24,25].

### 3.2. Clinical Utility of NfL in Multiple Sclerosis

**Figure 4:**
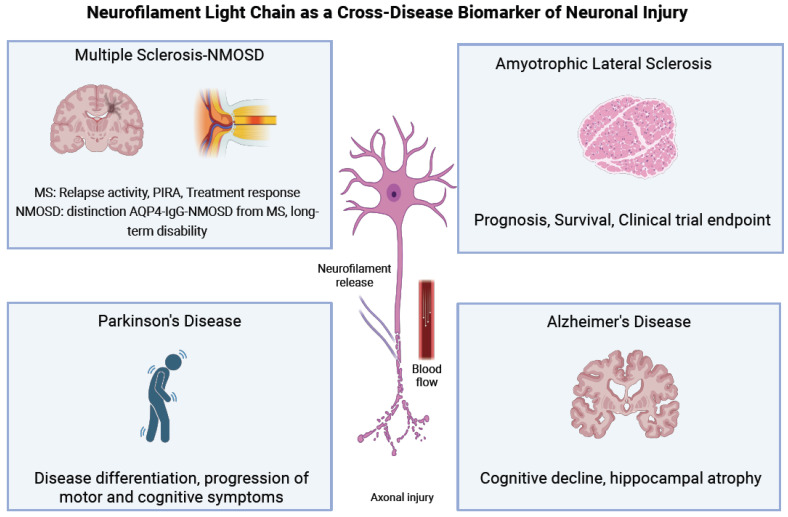
Neurofilament light chain (NfL) as a cross-disease biomarker of neuronal injury. Schematic representation of NfL release following axonal injury and its role as a sensitive but non-specific biomarker across major neurological diseases. Axonal damage leads to the release of NfL into the cerebrospinal fluid and subsequently into the bloodstream. Elevated NfL levels correlate with disease activity and progression in multiple sclerosis (MS) (relapse activity, progression independent of relapse activity [PIRA], and treatment response), amyotrophic lateral sclerosis (ALS) (prognosis, survival, and clinical trial endpoints), Alzheimer’s disease (AD) (cognitive decline and hippocampal atrophy), and Parkinson’s disease (PD) (disease differentiation and progression of motor and cognitive symptoms). This highlights the potential of NfL as a shared biomarker of neuroaxonal injury across diverse neurological conditions and as a biomarker in amyotrophic lateral sclerosis.

The clinical utility of NfL as a biomarker in MS has been established across various domains, including diagnosis, disease monitoring, and therapeutic decision-making. NfL is now recognized not only as a reflection of acute inflammatory damage but also as a dynamic measure of ongoing neurodegeneration [24] (Figure 4).

#### 3.2.1. The Use of sNfL in MS Diagnosis

NfL concentrations are significantly elevated in MS patients compared with healthy controls. In patients with RRMS, NfL levels typically increase during or shortly before clinical relapses, with sNfL peaking around 3–4 weeks after symptom onset and remaining elevated for the next 6–12 months [40,41]. Additionally, MRI studies consistently show strong associations between sNfL levels and radiological findings [24,34]. In particular, it has been demonstrated that gadolinium-enhancing lesions (Gd+) and new/enlarging lesions were independently associated with increased sNfL levels (17.8% and 4.9% increase per lesion, respectively; *p* < 0.001) [42].

The use of NfL alone, without additional clinical information, is not sufficient for diagnosing MS or for differentiating MS from other neuroinflammatory disorders. However, the measurement of sNfL in specific clinical contexts can help identify individuals who are at high risk for developing MS and also distinguish MS from post-infectious or monophasic demyelinating syndromes, where NfL levels are significantly lower [43].

Many studies have investigated the value of NfL for the diagnosis of MS at the time of the first demyelinating event. The presence of OCB (oligoclonal bands in the CSF) and/or Gd+ lesions are highly significant variables that help diagnose MS; they are included in the criteria of MS and are established risk factors of future relapses in MS [44]. Their prediction accuracy (sensitivity 72%, specificity 76%, and accuracy 79%) was increased (sensitivity 73%, specificity 79%, and accuracy 84%) by including the 90th percentile of sNfL in addition to the above two variables. Thus, when sNfL concentrations are especially high (>31 pg/mL) at the time of first symptoms, they are indicators of ongoing chronic CNS neuroinflammation. NfL may be useful for the diagnosis of MS, for the differentiation from clinically isolated syndrome (CIS), and in the future for the refinement of the McDonald criteria [45,46].

Importantly, NfL can also serve as a predictive biomarker. A longitudinal study that used a serum biobank with detailed clinical and demographical data demonstrated that sNfL levels were elevated approximately 6 years (with a range of 4 to 10 years) before disease onset and final diagnosis of MS [47]. Such studies on pre-symptomatic MS patients highly depict the extended prodromal phase of the disease before the initial clinical symptoms. Additionally, studies on patients with radiologically isolated syndrome (RIS) demonstrated that elevated sNfL levels suggest a higher risk of developing CIS or full-blown MS in the future [30,48,49].

The use of new innovative platforms using high-throughput chemiluminescent immunoassays, such as the Siemens Atellica^®^, gives us the opportunity to measure NfL levels with high precision and reproducibility. This NfL essay is a novel way to access critical, early insight into MS disease activity, and it is the first CE-marked blood test in Europe that offers fast, reliable, and holistic insight into patients’ MS disease activity. It is an approved prognostic risk assessment test setting a new standard, with a clear decision threshold [23,30]. A serum NfL cutoff of 12.9 pg/mL has been suggested as a clinically meaningful threshold in MS. NfL levels over this threshold are associated with worse prognosis concerning the occurrence of MRI lesions, brain or spinal atrophy, and long-term disability progression [25].

#### 3.2.2. The Use of NFL in Disease Monitoring

Across multiple large-scale studies, sNfL levels consistently correlate with relapse activity, MRI lesion load, Expanded Disability Status Scale (EDSS) progression, and brain atrophy rates. NfL provides a real-time measurement of neuronal damage, with levels rising in response to injury and remaining elevated for about 3 months [22]. On the other hand, conventional measures like MRI provide only a retrospective review. Importantly, changes in sNfL often precede clinical or radiological evidence of disease activity, underscoring its potential as an early-warning biomarker for subclinical progression [42,45,50].

A recent systematic review of 75 studies supported sNfL as a sensitive biomarker for monitoring both inflammatory and neurodegenerative aspects of MS. Higher sNfL levels were consistently associated with increased risk of relapses, faster disease progression, and greater radiological activity. On the other hand, lower levels predicted a higher likelihood of achieving no evidence of disease activity (NEDA) [22].

Several prospective studies have examined the potential of NfL at baseline to predict short-term and long-term outcomes among patients with MS [45]. In the short term, patients with sNfL levels above the 80th percentile are more likely to experience disability worsening, relapses, and accelerated brain atrophy within a year. This predictive ability remained significant also after adjusting for lesion load on MRI, showing the independent prognostic value of NfL. Over the long term, increased baseline NfL levels predict greater brain volume loss over 5–10 years. A recent meta-analysis that included more than 4000 patients further demonstrated that individuals with high sNfL levels reached an EDSS score of ≥4 significantly sooner than those with lower levels, although the time to relapse did not differ between the groups [51]. However, the overall predictive value of NfL levels for disability progression appears less consistent across the literature, possibly because chronic neurodegeneration can be masked by acute inflammation in MS [45,52].

Moreover, most available studies were not able to distinguish whether worsening outcomes reflected relapse-related injury or axonal injury from progressive disease. It is important to note that serial sNfL assessments exhibit stronger associations than baseline sNfL with brain atrophy and disability worsening. However, MS participants with serial sNfL during follow-up consistently in the reference range still exhibited accelerated brain atrophy compared to healthy controls. Thus, sNfL does not tell us the whole story, and its combination with other biomarkers may be more informative [19].

Furthermore, sNfL levels can be useful in recognizing pseudorelapses from real relapses. Most of the time, pseudorelapses are characterized by transient worsening of existing neurologic symptoms due to stressors like heat, exertion, or infection without reflecting an active neuroaxonal injury process [53]. Thus, a normal or at least steady level of NfL may help clinicians avoid the overdiagnosis of relapses and also avoid unnecessary corticosteroid use or change in DMTs.

Beyond clinical and radiological outcomes, the association of sNfL with subclinical cognitive or neuropsychiatric characteristics, including fatigue, depression, and processing speed deficits, is less systematically investigated and also conflicts in available data. In some studies, no or weak associations have been found [54], while others concluded that higher levels of sNfL were associated with poor processing speed or cognitive deterioration [55,56]. Those contradictory results show that larger and longer-term studies are necessary to clarify its utility in this setting.

#### 3.2.3. NFL as a Biomarker of Treatment Response

NfL levels consistently respond to a range of disease-modifying therapies (DMTs), making them an effective tool for monitoring therapeutic response. NfL levels of MS patients under immunomodulatory treatment are significantly lower when compared to untreated patients [18,57,58,59,60].

For example, ocrelizumab-treated patients with RRMS show a rapid and sustained reduction in sNfL over 12 months. At three months, NfL levels have already dropped significantly, coinciding with reductions in relapse rates and radiologic activity. By 12 months, 70% of patients had achieved “no evidence of disease activity” (NEDA-3), indicating clinical and radiologic remission alongside normalized NfL levels [61]. Similarly, natalizumab therapy in both adult and pediatric MS patients has been associated with significant reductions in CSF NfL concentrations. In pediatric-onset MS (POMS), natalizumab-treated patients exhibited a 50–60% reduction in CSF NfL within 6 months as well as clinical stabilization and decreased MRI activity [62]. These studies demonstrated the significant role of NfL in monitoring immunological response to therapy in younger populations [61,62]. Although most patients experience decreased NfL in response to therapy, NfL levels can remain elevated in 10–20% of patients despite high-efficacy treatment. This is possibly due to ongoing axonal injury from non-relapsing progressive disease (PIRA), which is less affected by current DMTs.

NfL levels can also differentiate therapies at a group level. In general, more significant reductions in NfL levels are seen in patients escalating to higher-efficacy therapies, whereas smaller changes are seen in those on milder treatments (*p* < 0.05) [60]. Stability of NfL levels is observed when patients switch between DMTs of similar efficacy. These findings suggest that monitoring sNfL changes longitudinally, rather than relying on single measurements, may better reflect disease activity and offer a promising approach to evaluate the impact of therapy on inflammatory activity in MS [46].

Baseline levels have also been shown to predict future therapy changes and escalations. Registry data from 1,261 Swedish patients with RRMS starting novel DMTs concluded that the choice of DMT is significantly associated with the degree of sNfL reduction. It also demonstrated specific patterns. Patients on alemtuzumab had the greatest reduction and lowest on-treatment sNfL, while patients who started teriflunomide had smaller decreases and higher on-treatment sNfL levels [60]. Additionally, phase 3 trials of fingolimod, natalizumab, and alemtuzumab demonstrated that sNfL changes mirrored clinical and MRI outcomes, supporting its potential use as a study endpoint. Consequently, subsequent studies opted for the use of sNfL as an endpoint in phase 2 studies [63,64]. The ASCLEPIOS trial, which compared ofatumumab with teriflunomide, moved forward with the use of sNfL prospectively as a secondary endpoint. It revealed significant differences in sNfL between treatment arms despite similar brain atrophy rates. These results provide further support for the use of longitudinal sNfL assessments and indicate that such approaches could help detect treatment effects not observed with conventional MRI metrics. In fact, sNfL levels are already a secondary outcome measure used in the majority of current investigational pharmaceutical clinical trials.

#### 3.2.4. NfL as Biomarker of Subclinical Disease Activity

NfL levels may guide initiation, de-escalation, or optimization of therapy in patients who do not meet clinical criteria for switching but show biochemical signs of non-response and ongoing axonal damage. At an individual level, longitudinally monitoring NfL levels can help detect suboptimal treatment response and individualize therapeutic decisions before overt clinical deterioration [46]. Importantly, sNfL elevations can also reveal subclinical disease activity in patients who appear clinically and radiologically stable. In the phase II study, APLIOS, patients who developed new Gd+ lesions at week 4 showed rising sNfL levels in the preceding month [65]. These findings suggest that sNfL can capture disease activity missed by infrequent MRI scans.

### 3.3. Prognostic and Therapeutic Implications in MS Subtypes

The ability of NfL to reflect both inflammatory and neurodegenerative components of disease is especially valuable for tailoring therapeutic strategies for different MS subtypes and different disease stages.

#### 3.3.1. Secondary Progressive Multiple Sclerosis (SPMS)

After 10–15 years of disease, RRMS may progress to SPMS, a phase characterized by gradual neurological decline with or without relapses. In this subtype, NfL functions primarily as a marker of neurodegeneration. NfL levels in SPMS are generally lower than in active RRMS but remain elevated compared to healthy controls, indicating ongoing axonal injury [66]. In general, though, direct comparisons between active RRMS and SPMS are less consistently quantified.

A rise in sNfL during late RRMS may precede clinical conversion to SPMS for 1–2 years. Subtle increases in NfL in patients who are otherwise relapse-free and radiologically stable may reflect underlying axonal damage not visible on imaging [14]. While anti-inflammatory drugs may still reduce NfL in SPMS patients with superimposed relapses, persistently elevated NfL in relapse-free SPMS suggests the need to shift toward neuroprotective or remyelinating strategies [14].

#### 3.3.2. Primary Progressive Multiple Sclerosis (PPMS)

Approximately 15% of patients with MS present from the start with a progressive form. This subtype is called PPMS and is characterized by steady neurological worsening from disease onset, typically without relapses. sNfL levels in PPMS are typically lower than in RRMS but are still moderately elevated compared to healthy controls (often 1.3–1.5× healthy control levels). In this context, NfL provides insight into chronic and slow non-inflammatory axonal damage. In PPMS, NfL is associated more with spinal cord atrophy and cortical thinning than with lesion count. This makes NfL particularly useful in a disease subtype where MRI shows fewer enhancing lesions [67]. In the ORATORIO trial assessing ocrelizumab in PPMS, sNfL levels fell by approximately 16% over 96 weeks in the treatment group, compared to only a 0.2% decrease with placebo, suggesting that sNfL can serve as a pharmacodynamic marker even in slow progressive disease [68].

#### 3.3.3. Pediatric-Onset MS (POMS)

POMS refers to MS with onset before the age of 18 and accounts for approximately 3–5% of total MS cases [62]. POMS is often associated with higher relapse rates and greater MRI lesion burden, but with a slower initial accumulation of disability compared to adult-onset MS. NfL levels are markedly elevated during relapses, when compared to healthy pediatric control values. Higher NfL levels were also found in clinically and radiologically stable POMS patients [69].

Additionally, elevated NfL levels in POMS patients help distinguish true MS from post-infectious or monophasic demyelinating syndromes, where NfL levels are significantly lower [43]. This is especially valuable since children often present with atypical or polysymptomatic features. It is also important to note that establishing age-adjusted values is critical for interpreting NfL in pediatric populations, as normal NfL levels are inherently higher in children due to developmental axonal turnover.

### 3.4. Integration with Other Biomarkers

The prognostic value of NfL can be enhanced through a combination with complementary markers. A recent study that measured 24 fluid biomarkers, including NfL, demonstrated that combinations of ≤6 CSF or serum biomarkers significantly improve the prediction of MS prognosis, compared to measuring NfL alone [70]. The biomarker osteopontin, which is a cytokine expressed by various cell types, including immune cells, showed the highest fold difference after NfL and possibly adds an immune-process insight in combination with NfL [70].

Glial fibrillary acidic protein (GFAP), which is a marker of astrocytic activation, has gained significant research attention as a second major blood biomarker. In comparative analyses, NfL was more sensitive in detecting active inflammation and treatment response in MS, while GFAP better reflected chronic progression, making it a valuable marker for ongoing glial-driven neurodegenerative pathology [71]. A combination of sNfL and GFAP measurements can help discriminate RRMS from SPMS, because GFAP is particularly elevated in SPMS and correlates with neuroinflammatory processes distinct from those captured by NfL [72]. Another astrolytic biomarker, like GFAP, is CSF chitinase-3-like protein 1 (CHI3L1). Even though it can reflect different pathological processes from GFAP, its clinical utility is limited by the need for CSF sampling [73].

Furthermore, the use of markers associated with B-cell activity can be quite informative, considering the contribution of B cells to MS pathophysiology. Oligoclonal bands or CSF CD80+/CD19+ ratios have shown potential for distinguishing disease courses in early MS [74]. Additionally, in contrast to sNfL, which reflects axonal damage of the white matter, pairing sNfL with markers of gray matter pathology, such as reduced CSF parvalbumin, may enhance the detection of slow neurodegenerative changes. Paralvulin is a protein expressed in gamma-aminobutyric acid (GABAergic interneurons), and its reduced levels have been correlated with meningeal inflammation and cortical lesion load [75]. Overall, multimodal biomarker strategies incorporating blood biomarkers as well as other useful tests like Optical Coherence Tomography (OCT) could provide a fuller view of MS biology but require validation in large cohorts.

### 3.5. Biomarkers for Neuromyelitis Optica Spectrum Disorders (NMOSD) and Myelin Oligodendrocyte Glycoprotein Antibody-Associated Disease (MOGAD)

Neuromyelitis Optica Spectrum Disorders (NMOSD) constitutes a spectrum of rare autoimmune diseases of the CNS marked by episodes of transverse myelitis, optic neuritis, and other demyelinating attacks. Despite recent therapeutic advances, there are no established guidelines on whom to treat with immunosuppressive medications and on the duration of treatment, as relapses can occur even after prolonged periods of remission. Therefore, clinically validated biomarkers are needed to guide care [76].

Although aquaporin-4 immunoglobulin G (AQP4-IgG) is central to NMOSD pathophysiology, it is not considered a useful biomarker for monitoring disease activity or treatment response [76]. Because NMOSD is primarily an astrocytopathy with secondary neuronal injury, GFAP and NfL are released into CSF during attacks and become measurable in serum. Their levels, in both serum and CSF, are elevated at baseline in NMOSD compared to healthy controls. Serum GFAP (sGFAP) is higher in AQP4-IgG-positive NMOSD when compared to both MS and to a lesser extent MOGAD, implying that sGFAP, but not sNfL, may aid in distinguishing NMOSD from other autoimmune CNS diseases [77,78]. Notably, sGFAP tends to be less elevated in AQP4-seronegative NMOSD, whereas sNfL is increased in both seropositive and seronegative NMOSD. These findings imply a unique pathology of seronegative NMOSD, likely not involving primary astrocyte injury [79].

Although many studies find higher sGFAP in AQP4-IgG-positive NMOSD than in MS, there is overlap in the concentrations reported that precludes a universally accepted diagnostic cutoff. However, the ratio of sGFAP/sNfL has shown promise for distinguishing AQP4-IgG-positive NMOSD from MS [76,77,78]. A ratio with values above 5.71 is 73% sensitive and 75.8% specific for AQP4-IgG-positive NMOSD compared to MS [77]. The timing of sampling, though, is critical for interpreting results. sGFAP declines more rapidly after relapse, while sNfL can remain high for months to years, indicating ongoing neuronal injury [77,78]. Most evidence suggests that sNfL is not a useful biomarker for predicting relapse. However, sNfL may better reflect long-term disability risk [80].

In myelin oligodendrocyte glycoprotein antibody-associated disease (MOGAD), sNfL has also emerged as a marker of acute disease activity. In both adults and children with MOGAD, sNfL levels are significantly higher during relapse compared to remission and healthy controls. sNfL levels were correlated with recent relapses, seizures, and brain lesions on MRI [81]. These findings indicate that sNfL may be useful for identifying active inflammation in MOGAD, with comparable performance across age groups.

## 4. Neurofilaments as Biomarkers in Amyotrophic Lateral Sclerosis

### 4.1. Introduction to Amyotrophic Lateral Sclerosis

Amyotrophic lateral sclerosis (ALS), also referred to as motor neuron disease (MND) or Lou Gehrig’s disease, is a fatal neurodegenerative disorder characterized by progressive degeneration of upper and lower motor neurons. ALS leads to muscle weakness and atrophy and ultimately results in total paralysis and death. About 90% of patients with ALS are classified as sporadic (sALS) and 10% as familial (fALS), with over 50 genes implicated [82]. The four most common genetic mutations occur in the SOD1, C9orf72, TARDBP, and FUS genes [83]. There is currently no known treatment able to arrest the course of ALS, and death typically occurs 3–5 years after symptom onset [84]. Within that framework, ALS research requires easy-to-use, validated biomarkers with significant clinical utility to enhance patient care and to expedite ALS treatment development.

### 4.2. Biomarkers in ALS

A plethora of candidate biomarkers has been investigated in clinical studies for ALS, reflecting the different pathophysiological processes in ALS, from protein aggregation to axonal degeneration. Several metabolomic and proteomic studies have been performed using different types of biofluids in the search for diagnostic and prognostic biomarkers [85,86]. To date, NFs are the most studied and most promising biomarker in the ALS field [11]. Initially, their measurement was limited to CSF. However, recent advances in immunoassay technologies have made it possible to quantify NFs in blood, serum, or plasma, maintaining high analytical sensitivity [84] (Figure 4).

### 4.3. Neurofilaments as Diagnostic Biomarkers

While elevated NF levels in biofluids are not disease-specific, they can support the diagnosis of ALS by confirming underlying neurodegeneration. Compared to healthy controls, both NfL and NfH are significantly elevated in the CSF and blood of ALS patients [11]. Both have high sensitivity and specificity in distinguishing ALS from disease mimics, as depicted in a large meta-analysis [87].

Importantly, NF levels are typically higher in ALS than in other neurological disorders, including frontotemporal dementia (FTD), Alzheimer’s disease (AD), and stroke, likely due to rapid loss and selective vulnerability of large, myelinated motor neurons, which leads to a mass release of NFs into CSF and blood [88,89]. NfL displayed the best diagnostic performance in discriminating ALS from healthy controls (area under the curve [AUC] = 0.990), other neurodegenerative disorders (AUC = 0.946), and ALS mimic disorders (AUC = 0.850) [88]. In comparison to other neurodegenerative diseases, sNfL levels in ALS are only exceeded by Creutzfeldt–Jakob disease [90]. Still, normal neurofilament levels do not exclude the diagnosis of ALS or other neurodegenerative diseases.

In clinical practice, the diagnostic sensitivity of sNfL ranges from 76% to 100%, with specificity between 75% and 92% [91,92,93,94]. Nevertheless, the cornerstone of diagnosis remains clinical expertise and appropriate neurophysiological testing. In specialized ALS clinics, the role of NfL is to reinforce clinical suspicion and help confirm or rule out ALS, particularly when levels are extremely high or low [93,94]. NF measurement can be especially helpful in distinguishing ALS from peripheral nervous system mimics (e.g., inclusion body myositis, multifocal motor neuropathy, etc. [95]) or in cases with delayed presentation and ambiguous chronology (e.g., benign focal amyotrophy, inflammatory plexopathy, and compressive radiculopathy) [94]. In one of the largest case series of MND mimics, the highest NF levels were observed in disorders of the PNS such as CIDP, myopathies, and polyneuropathies [1,96,97]. In comparison, MNDs with a slower progression, such as hereditary spastic paraplegia (HSP) [98], spinal and bulbar muscular atrophy [99], and possibly primary lateral sclerosis (PLS) [98], were associated with close to normal NF levels, a distinction that may aid in their differentiation from ALS [1]. While these findings are encouraging, they still require validation across laboratories and confirmation in larger patient cohorts.

### 4.4. Neurofilaments as Prognostic Biomarkers

It is well established that in many neurodegenerative diseases, elevated NF levels precede clinical symptom onset. The point at which symptoms emerge is termed clinical phenoconversion. Identifying the time window during which NfL levels begin to rise offers a valuable opportunity to predict disease onset and course.

Genetic forms of ALS provide a unique model to study presymptomatic individuals. The PRE-FALS (Pre-Symptomatic Familial ALS, NCT00317616) longitudinal study followed 84 asymptomatic mutation carriers, 34 controls, and 17 ALS patients (including 10 phenoconverters) [100,101]. NF levels (cNfL, sNfL, and CSF NfH) were initially indistinguishable between mutation carriers and controls. However, serial measurements revealed progressive increases in sNfL several months to years before phenoconversion, 6–12 months in SOD1 carriers, ≥2 years in FUS carriers, and up to 3.5 years in C9orf72 carriers [102].

Following clinical onset, NfL levels continue to rise until reaching a plateau, which tends to remain stable over the long term, possibly reflecting immune clearance or aggregation dynamics [103]. Notably, the absolute value at which the plateau is established varies across individuals [104]. NF concentrations at steady-state show promise as prognostic biomarkers, and their prognostic value is greatest when assessed early, before significant disease progression has occurred [105,106]. Additionally, clinical trial design could be facilitated by using NF levels for the stratification of ALS patients into balanced groups depending on disease aggressiveness [86]. Stratifying slow and fast progressors in different clinical treatment arms would improve group balance and clinical trial reliability [106].

Numerous studies have correlated higher NF levels with lower ALS Functional Rating Scale (ALSFRS-R) scores [27,90,107,108], faster progression [8,90,103,108,109,110], shorter survival [90,103,108,109,110], greater upper motor neuron burden [8,90,110,111], and bulbar onset [27,107,111], all indicative of worse prognosis [11]. The strongest prognostic associations have been observed with NfL, with somewhat weaker evidence for NfH [87].

### 4.5. Neurofilaments in Clinical Trials

#### 4.5.1. NFs in Clinical Trials as Predictive Biomarkers

Given the biological heterogeneity of ALS, there is growing interest in using biomarkers to tailor therapies to individual patients. NFs have shown particular promise in this regard, with the most compelling example coming from the VALOR clinical trial of Tofersen, an antisense oligonucleotide targeting mutant SOD1. Though SOD1 mutations account for only 20% of familial and ~3% of sporadic ALS, this subgroup was the first to benefit from a therapy targeting a specific pathogenic mechanism.

The administration of Tofersen led to significant reductions in CSF SOD1 protein and plasma NfL levels. Although clinical efficacy initially was modest, likely due to the heterogeneity of enrolled participants, later analysis and data from the extension phase (28 weeks during the VALOR study and 24 weeks during the open-label extension) showed stabilization of disease progression across endpoints and even improvement in function, respiration, and strength [85,112]. This trial established several milestones: the first genetic therapy for ALS, the first to show both disease slowing and functional improvement, and the first ALS drug to receive FDA accelerated approval based on NF reduction as a surrogate endpoint.

#### 4.5.2. NFs in Clinical Trials as Susceptibility and Risk Biomarkers

Building on the findings of the PRE-FALS and VALOR trial, the ATLAS trial (NCT04856982) represents a paradigm shift in ALS therapeutics. This ongoing randomized, placebo-controlled phase 3 trial enrolls asymptomatic adult SOD1 mutation carriers and uses longitudinal sNfL measurements to identify the optimal time to initiate Tofersen therapy, before phenoconversion [105]. A predefined sNfL threshold and trajectory model guide the intervention. If successful, this will mark the first example of biomarker-driven preventive therapy in ALS.

The rise in NF levels before ALS symptom onset is not limited to individuals with familial ALS. Notably, in a matched case–control study nested in three large prospective US cohorts (the Nurses’ Health Study, the Health Professionals Follow-Up Study, and the Multiethnic Cohort), sNfL was increased within 12–24 months prior to sporadic ALS diagnosis of participants [113]. These observations attest to the potential utility of sNfL as an early biomarker to detect presymptomatic individuals approaching phenoconversion. However, considerable ethical considerations are raised regarding early presymptomatic diagnosis and risk disclosure.

#### 4.5.3. NFs in Clinical Trials as Pharmacodynamic Biomarkers

A robust correlation of sNfL levels with disease progression and survival in ALS has repeatedly been reported. Their relative longitudinal stability within individuals, alongside their predictive value for progression and survival, makes them a promising biomarker endpoint for clinical trials. As a result, NFs are being used in many clinical trials as secondary endpoints, and even primary endpoints, in order to signal the existence of biological activity of an experimental therapy and to quickly assess if a drug is successful or not. Many phase II trials now include sNfL monitoring in order to accelerate the acquisition of results and the decision to advance or not to phase III trials, where clinical efficacy must be demonstrated [104,106].

In a simulation study, it was demonstrated that incorporating baseline sNfL levels as covariates in ALS clinical trials could reduce required sample sizes by 8.2% [114]. Notably, comparable results were found in a recent meta-analysis including 60 studies and 8801 participants [87]. Furthermore, using sNfL as a pharmacodynamic biomarker may enable even greater reductions compared to traditional phase 2 trial designs that rely on changes in ALSFRS-R as the primary endpoint [1,114].

### 4.6. Combination of NFs with Other Biomarkers

The entire range of pathology in ALS is not captured by NfL alone. For instance, NfL levels do not accurately reflect other prominent pathological characteristics of ALS, like inflammation and vascular damage. In order to provide a more complete picture of disease mechanisms, NfL could be combined with other biomarkers.

A recent study combined CSF NfL with CSF intercellular adhesion molecule-1, a marker of vascular damage, and with serum interferon gamma, a marker of peripheral inflammation. This combination led to better discrimination of patients with ALS from patients with inflammatory peripheral neuropathies than when using CSF NfL alone [115]. A more comprehensive understanding of ALS could be attained by combining these different biomarkers, which would enhance diagnostic precision, prognostic predictions, and treatment response monitoring [116].

Better patient outcomes may result from this biomarker combination approach, which may be especially helpful in capturing the complex nature of ALS. Moreover, the integration of NF data with genetic, clinical, digital, imaging, and other molecular biomarkers, combined with advances in machine learning, is expected to enhance the accuracy and efficiency of ALS diagnosis, prognosis, and treatment. Finally, before NfL can be considered a viable tool for population-wide screening or early therapeutic intervention in ALS, its cost-effectiveness, optimal sampling frequency, and predictive value in asymptomatic individuals must be rigorously established through prospective studies.

## 5. Neurofilaments in Parkinson’s Disease

### 5.1. Parkinson’s Disease

Parkinson’s disease (PD) is the second most common neurodegenerative disease. According to the UK Brain Bank’s criteria, PD involves motor symptoms including bradykinesia, rigidity, rest tremor, and gait deterioration in later stages. Non-motor symptoms include olfaction deficits, REM sleep behavior disorders (RBD), constipation, and depression. Such symptoms may predate motor involvement. In later stages of cognitive deterioration, psychotic features and autonomic dysfunction might also emerge. In terms of pathology, PD is defined by the progressive degeneration of dopaminergic neurons in the substantia nigra, resulting in a reduction in dopamine neurotransmission. Lewy bodies, a hallmark of PD, are aggregates of various proteins, including α-synuclein, NF proteins, ubiquitin, proteasome subunits, torsin A, and parkin [117].

### 5.2. Nf Dysfunction in PD

NF dysfunction in PD is linked to several molecular mechanisms that contribute to neuronal degeneration [84]. There are numerous key pathways involved: (a) Protein aggregation and misfolding. NFs, like other cytoskeletal proteins, can become hyperphosphorylated, a process that in turn leads to abnormal aggregation. In PD, alpha-synuclein interacts with NFs, disrupting their normal function and contributing to Lewy body formation. (b) Impaired axonal transport. NFs play a major role in maintaining axonal integrity. In PD, dysfunctional NFs can impair axonal transport, leading to reduced trafficking of essential proteins and organelles in neurons. This disruption contributes to neuronal death and disease progression. (c) Oxidative stress and NF damage. Oxidative stress is a crucial factor in PD pathogenesis. Reactive oxygen species (ROS) can damage NFs, leading to structural instability and impaired neuronal function. This oxidative damage further triggers neurodegeneration. (d) Mitochondrial dysfunction. Mitochondrial dysfunction is a key element of PD. NFs interact with mitochondria, and their dysfunction can lead to energy deficits, attenuating neuronal survival. Mitochondrial damage also increases oxidative stress, aggravating NF degradation. (e) Autophagy and protein clearance defects. NF degradation is partially controlled by autophagy and proteasomal pathways. In PD, these mechanisms are inadequate, leading to the accumulation of NF aggregates and contributing to neuronal toxicity. (f) Genetic mutations affecting NF regulation. Certain genetic mutations associated with PD, such as LRRK2 and PINK1, have been linked to NF dysfunction. These mutations influence phosphorylation and degradation of NFs and result in disease progression.

Reduced levels of NfL and NfH mRNA have been observed in PD. An investigation of the proteins in substantia nigra samples from individuals with PD revealed a decrease in the levels of NfM and NfL proteins in the brain. Additionally, it has been found that there is an increased presence of an oxidative-state cellular environment. The levels of NfL mRNA are reduced in proportion to the clinical severity of PD [118,119].

A genetic link between NFs and Parkinson’s disease has been previously described. Han and his team conducted a second screening on 102 individuals with PD and 45 healthy controls to look for a specific mutation (G1747A) in the NEFM gene [120,121], indicating that mutations in the gene may contribute to the vulnerability to PD. In a published case report, a young patient (16 years old) experiencing PD symptoms had a specific genetic mutation in the NEFM gene. This mutation affected the coding area responsible for the rod domain 2B of the NfM protein [122]. The alteration in the base pair led to the substitution of glycine with serine at position 336, which has been suggested to interfere with the formation of NF structures.

### 5.3. NFs as a Biomarker in Parkinson’s Disease

A biomarker represents a characteristic trait that is objectively measured and evaluated as an indicator of normal biological processes, pathogenic processes, or pharmacological responses to a treatment. Biomarkers can be any physical characteristic linked to the presence of disease (diagnostic marker) or any characteristic that changes over time in a way that can reflect the disease progression (progression marker). Since the clinical diagnosis of PD is usually possible only after a significant number of dopaminergic neurons in the substantia nigra have degenerated, there is a need for biomarkers of risk or susceptibility for developing the disease and biomarkers specific to the preclinical or premotor stage. A wide range of candidate markers have been evaluated for PD, including olfactory testing, neuroimaging, tissue, blood, and CSF analysis, and genetic testing [84].

Fluid biomarkers (blood and CSF) with NfL offer valuable and easily accessible tools (Figure 4). Elevated levels of NfL in blood and CSF are frequently observed in neurodegenerative disorders like AD, ALS, and Parkinsonian syndromes. The significance of NfL is shedding light on its roles in pathology, diagnostic value, and implications for patient care.

### 5.4. Potential Applications of NFs Early in Pd Diagnosis

NfL levels could assist in detecting PD before clinical symptoms become severe. Regarding monitoring disease progression, changes in NF levels may provide insight into the rate of neuronal degeneration. Additionally, in the scope of precision medicine, NfL as a biomarker may help tailor therapies based on disease severity and prognosis. Further research is needed to establish standardized thresholds for clinical use.

Recent studies reported that CSF and plasma NfL levels were lower in PD but elevated in atypical PD subtypes like MSA and PSP, aiding in distinguishing diseases with similar symptomatology [123,124]. However, elevated NfL concentrations predict PD-associated dementia (PDD), a fatal disease manifestation compared to PD without dementia [125,126].

A study attempted to quantify NfL levels in serum using an ultrasensitive assay such as ELISA. The findings show that the mean baseline sNfL increased significantly in PD compared to the controls. Specific cognitive scores showed a negative correlation, and motor scores positively correlated with NfL [127].

### 5.5. Combination of NFs with Other Biomarkers in Pd

The prognostic roles of sNfL, phospho-tau, beta-amyloid, and GFAP are still debated in PD. A recent study by Pilotto et al. assessed the role of these biomarkers in PD progression prognosis [128]. It appears that NfL and GFAP correlated with baseline motor and non-motor severity measures. At follow-up, NfL emerged as the best predictor of progression with a marginal effect observed for GFAP and p-tau181.

## 6. Neurofilaments in Alzheimer’s Disease

### 6.1. Alzheimer’s Disease

Alzheimer’s disease (AD) represents a major cause of dementia and affects up to 25% of people during their lifespan. Clinical features include mainly an amnestic phenotype with short-term memory impairment. More bizarre phenotypes can be observed, especially in early-onset manifestation. These include an apraxia–aphasia syndrome, logopenic AD, behavioral variant AD, posterior cortical atrophy, and corticobasal degeneration-like syndrome.

Neurofibrillary tangles (mainly formed by Tau protein and NFs) are a pathological hallmark of AD, along with irregular alterations of proteins, including tau, amyloid-β, NFs, ubiquitin, and other proteins that compose the cytoskeleton. Amyloid beta is formed during the breakdown of the protein encoded by the APP gene, and two main forms have been described: Aβ42 and Aβ40. Aβ42 represents < 10% of the total but is more toxic. The involvement of senile plaques and neurofibrillary tangles in AD is currently the subject of intense controversy. The prevailing hypothesis is that the disease is triggered by the accumulation of protein in different polymeric and monomeric forms. On the other hand, other researchers suggest that this protein accumulation is a reaction to the initial damage.

Protein buildup is considered to be a reaction to oxidative damage that occurs as a result of aging. Thus, the prevention of oxidative damage could be a beneficial intervention to reduce NF protein abnormalities. Previous research indicates that tau protein and NF proteins present in neurofibrillary tangles undergo significant phosphorylation due to an imbalance in the activity of kinases and phosphatases [129].

The gene expression levels of NF proteins undergo structural alterations in AD. Increased oxidative stress markers are present in both NF proteins and AD lesions [130]. A study investigated the occurrence of NF proteins and their phosphorylation sites in the neurites of senile plaques. The results showed the existence of unidentified phosphorylation sites on NfL. The authors propose that NFs may play a significant and intricate function in developing senile plaques [131].

Sonoda and colleagues have shown that the phosphatase and tensin homolog on chromosome 10 (PTEN), which is associated with AD, acts as both a tyrosine phosphatase and a lipid phosphatase. Furthermore, it exists as abnormal tau and phosphorylated NF proteins in neurons. These findings indicate that the reorganization of PTEN to neurotic pathology may play a crucial role in the growth of tau and NF pathology in the brains of individuals with AD [132].

### 6.2. NFs as Biomarker in Alzheimer’s Disease

The role of NFs as a putative biomarker in AD is increasingly recognized. Axonal loss and demyelination are correlated with loss of white matter integrity in AD. Increased NfL levels in blood and CSF indicate neurodegeneration [133] (Figure 4). Furthermore, according to former research, there is a significant association between CSF and sNfL [134] in AD patients. The findings suggest that NfL levels compared to controls are higher in AD dementia than in individuals with mild cognitive impairment due to AD [134,135,136].

As far as memory is concerned, impaired white matter integrity can affect the connections between regions involved in memory formation and recovery, such as the hippocampus and prefrontal cortex. A recent study investigated the relationship between blood NfL levels and white matter integrity in autosomal dominant AD individuals. Elevated NfL levels in mutation carriers were linked to greater white matter hyperintensity volume. Alterations in diffusion tensor imaging showed decreased white matter integrity compared to non-carriers [137].

The Ontario Neurodegenerative Disease Research Initiative assessed various biomarkers in multiple forms of neurodegeneration. Glial fibrillary acidic protein (GFAP), neurofilament light chain (NfL), phosphorylated tau (p-tau)181, and amyloid beta (Aβ)42/40 were measured using ultra-sensitive SIMOA immunoassays in 44 healthy controls and 480 participants diagnosed with AD/MCI (mild cognitive impairment), Parkinson’s disease (PD), frontotemporal dementia (FTD) spectrum disorders, or cerebrovascular disease (CVD). GFAP, NfL, and/or p-tau181 were elevated among all diseases compared to controls. Increased levels of both plasma GFAP and NfL were found to be broadly associated with worse outcomes in most baseline cognitive domains in the pooled cohort of all diseases. This effect appeared to be mainly driven by the AD/MCI and PD cohorts, in which similar associations were found. While GFAP, NfL, and p-tau181 were highly predictive across diseases, p-tau181 was more specific to the AD/MCI cohort [138].

An additional study explored the relationship between CSF NfL concentration and AD progression. It found that higher CSF NfL levels correlated with cognitive decline, hippocampal atrophy, and white matter changes, suggesting its utility in monitoring disease severity [139].

Moreover, Freudenberg-Hua and co-authors investigated the association of the established high-effect variants for AD in APOE and TREM2 with plasma levels of protein biomarkers, GFAP and NfL, using data from over 50,000 participants from the UK Biobank (UKB) [140]. APOE4 was associated with elevated levels of plasma GFAP, and to a lesser extent, NfL. In contrast, the protective APOE2 allele showed no effect on GFAP or NfL. It appears that major genetic risk factors for AD differentially affect dementia protein biomarkers across age, indicating gene-specific pathways with potential therapeutic implications.

Findings of previous studies indicated that higher NfL levels were linked to faster cognitive decline, reinforcing its potential as a prognostic biomarker. These studies reinforce the growing interest in NfL as a biomarker for AD, offering insights into disease mechanisms and potential applications for early diagnosis and treatment monitoring.

## 7. Neurofilaments in Frontotemporal Dementia and Atypical Parkinsonism

Frontotemporal dementia (FTD) is associated with speech and behavioral problems along with dementia. Different subtypes of FTD, including progressive non-fluent aphasia (PNFA), progressive aphasias, and semantic dementia (SD), exhibit distinct levels of NfL [141]. The gradual deterioration of neurons in the brain’s frontal and temporal areas is a hallmark of FTD, typically exhibiting an earlier onset than other forms of dementia.

A recent study reported that NfL levels are higher in FTD individuals than in controls and AD individuals. Compared to healthy controls and AD, the highest concentrations are noted in SD and behavioral variant (bvFTD), while NfL levels are lower in PNFA. However, statistical differences among the subtypes’ NfL levels were not observed [142]. Another research group reported that NfH levels are high in FTD patients, and inherited variants of FTD have elevated NfL levels in comparison to controls [143].

Moreover, a recent study by Sheth et al. evaluated plasma NfL and GFAP longitudinally across FTD spectrum disorders. Compared to controls, GFAP and NfL were elevated in each FTD syndrome, but GFAP, unlike NfL, poorly discriminated controls from participants with mild symptoms. Similarly, both baseline GFAP and NfL were higher in presymptomatic mutation carriers who later phenoconverted, but NfL better distinguished non-converters from phenoconverters. GFAP and NfL were associated with disease severity indicators and survival, but NfL far outperformed GFAP [144].

NfL consistently outmatched GFAP as a prognostic and predictive biomarker for participants with FTD syndrome, and as a susceptibility/risk biomarker for people at genetic risk of FTD. Such results pave the way for the clinical use of NfL as a biomarker for frontotemporal spectrum disorders.

## 8. Limitations in the Use of Neurofilaments and Future Directions

Before NfL is widely used in standard clinical practice, several limitations must be recognized, despite the mounting evidence of its clinical utility in MS, ALS, and other neurodegenerative diseases. The absence of global standardization in reference materials and assay procedures is one of the main obstacles. SIMOA and chemiluminescence assays are used to reliably measure NfL levels in blood, even at low concentrations, with high sensitivity [84]. However, variations in sensitivity, accessibility, and method standardization may affect the results and their clinical applicability.

Furthermore, there is a lack of universally accepted normative reference ranges. There are ongoing efforts to harmonize interpretation and reference ranges across laboratories and populations. Experts have called for international consensus on sample handling protocols, assay cutoffs, and population-specific normative data [25]. The clinical interpretation of NfL will benefit greatly from defining what constitutes normal, borderline, or pathological values based on age, disease phenotype, and treatment status.

Although age-adjusted cut-off values have been proposed based on population studies [145], these were limited to individuals aged 38–85 years, leaving significant gaps in our understanding of NfL levels and dynamics in younger adults. Additionally, for individuals aged > 60 years, substantial variability in sNfL was found, likely reflecting not only aging but also comorbid conditions and other subclinical CNS pathology, such as stroke, traumatic brain injury, or transient ischemic attack [146,147]. NfL measurements should thus always be evaluated in the context of a thorough clinical assessment. The need for large, multicenter cohort studies to define NF reference values across diverse populations is highlighted by the fact that other potential sources of variation, such as sex, ethnicity, and comorbid conditions, have not been thoroughly addressed in reference datasets.

An important limitation is that different immunoassay platforms (e.g., ELISA, ECLIA, SIMOA, and Atellica) do not yield identical absolute NfL values, which complicates direct comparison across studies. While all platforms show strong relative correlations, cutoffs established for one assay cannot be directly transferred to another. This highlights the need for assay-specific reference ranges and, when possible, longitudinal follow-up of individual patients on the same platform. Conversion models have been proposed, but these approaches require further validation before being implemented in clinical practice. Generally, borderline elevations of sNfL should be interpreted with caution. In such cases, professionals should evaluate patients at an individual level with serial monitoring and observing how the levels fluctuate [148]. Patients therefore function as their own controls in this approach. However, it is important to recognize that in order to determine whether a change is clinically significant, we need to account for both biological and analytical variability. In healthy individuals, day-to-day variation is minimal (~3%). Week-to-week variation ranges from 7 to 10% and analytical variation from 6 to 7% [149,150,151]. Together, these studies define a reference change value (RCV) of approximately 23–31%, which means that only changes exceeding this threshold can be interpreted as a meaningful difference [149,150,152].

A helpful strategy to improve interpretability is the use of z-scores as opposed to absolute values for biomarkers [152]. When baseline sNfL values are unavailable, the alternative of age- and BMI-adjusted z-scores can be used. These z-scores or percentiles can be derived from large reference datasets, e.g., >5000 healthy controls in the Kuhle group database [33]. In that study, a z-score > 1.5 was associated with a more than threefold increased risk of future disease activity across the MS spectrum, including in patients with no current evidence of disease activity [33].

Particularly in MS, the use of z-scores helps to correct the variability within populations and adjust pathological cutoffs. Beyond NfL alone, combining z-scores of multiple biomarkers, such as NfL and GFAP, might increase sensitivity and specificity with regard to disease severity or relapse risk. With this strategy, a more individualized approach could be applied in MS [33,76]. Overall, standardization across assays and validation in large, multi-ethnic cohorts is crucial in order to establish robust and universally applicable cutoff values.

Furthermore, the implementation of NfL testing in clinical practice faces additional barriers that are related to cost and access. NfL quantification remains relatively expensive compared to routine blood tests and is not yet consistently reimbursed by insurers [153]. Nevertheless, routine sNfL testing has the potential to lower overall healthcare costs by reducing unnecessary MRI use and preventing prolonged treatment with suboptimal DMTs [152]. Overall, as interest in NfL grows, the development of assays on existing laboratory platforms is expected to reduce costs and improve accessibility. Toward this direction, apart from well-established measurement methods like SIMOA, novel measurement applications for NfL levels in specific neurological disorders, like Siemens Atellica in MS, with the capability of measuring many samples in one run, within a cost-effective framework, have advanced their everyday utility to another level, making this important biomarker available for a wider range of users and laboratories [30,86].

## 9. Conclusions

In summary, NFs, and especially NfL, have moved from being observational biomarkers reflecting axonal injury to actively shaping clinical research and practice. In MS, they are increasingly used to monitor treatment response and silent disease activity, complementing MRI and clinical measures. In ALS, the use of NFs has progressed even further by becoming a pharmacodynamic endpoint in therapeutic trials, and, also, NFs are being discussed by regulatory bodies as potential drug development tools. ALS may thus serve as a prototype condition where NFs demonstrate their full translational potential, while MS illustrates their value in clinical management. Beyond ALS and MS, elevated NF levels have consistently been observed in other neurodegenerative diseases, such as AD, FTD, PD, and atypical Parkinsonian syndromes, highlighting their broad applicability as a marker of neuroaxonal injury, though with limited disease specificity.

The key innovation of this review is its integration of evidence across both inflammatory and neurodegenerative conditions, combined with a critical appraisal of established and novel analytical platforms, assay harmonization, and confounding factors. NFs are not only a biomarker of neuronal injury but also a candidate driver of precision medicine, enabling individualized monitoring and stratification of patients.

Nevertheless, several barriers must be addressed before routine adoption is possible. These include assay standardization across platforms, establishment of universally applicable reference ranges, careful adjustment for biological variability, and demonstration of cost-effectiveness in clinical workflows. Moving forward, major advances are expected to come from combining NFs with other biomarkers reflecting different pathological processes, but also with advanced imaging, digital tools, and artificial intelligence (AI) algorithms. Ultimately, the integration of NFs into multimodal biomarker frameworks may transform the care of patients with neuroautoimmune and neurodegenerative diseases, bridging the gap between biology, clinical practice, and therapeutic innovation.

## Figures and Tables

**Table 1:** Analytical platforms for NfL quantification.

Platform	Principle	Sample Type	Advantages	Limitations	Clinical Applicability
**Enzyme-linked immunosorbent Assays (ELISA)**	Antibody-based detection	Mainly CSF	Established method; low cost; significant accuracy	Low sensitivity for blood; labor-intensive; research use only	Research: limited clinical use
**Electrochemiluminescence (ECLIA)**	Luminescence generated by the electrochemical reactions of antibodies	CSF and serum	Semi-sensitive; partially automated	Less sensitive than SIMOA; moderate throughput	Emerging clinical utility
**Single Molecule Array** **(SIMOA)**	Ultra-sensitive bead-based digital immunoassay	CSF and serum	Ultrasensitive; Detects sub-pg/mL levels; strong CSF–serum correlation; high reproducibility	Higher cost; requires specialized platform	Widely used; FDA Breakthrough Device designation
**High-Throughput Chemiluminescent Immunoassays**	Automated chemiluminescent detection	Serum (routine)	Full automation; scalable; robust reproducibility	Limited availability; requires large clinical analyzers	Clinical integration (e.g., Siemens Atellica^®^); the first CE-marked blood test in Europe
**Emerging Technologies**	Immunoprecipitation with mass spectrometry	CSF and tissue	Identifies NfL proteoforms; potential CNS vs. PNS distinction	Currently research only; technically complex	Future personalized biomarker assays

## Data Availability

No new data were created or analyzed in this study.
